# Combating antimicrobial resistance: the silent war

**DOI:** 10.3389/fphar.2024.1347750

**Published:** 2024-02-14

**Authors:** Letao Bo, Haidong Sun, Yi-Dong Li, Jonathan Zhu, John N. D. Wurpel, Hanli Lin, Zhe-Sheng Chen

**Affiliations:** ^1^ Department of Pharmaceutical Sciences, College of Pharmacy and Health Sciences, St. John’s University, Queens, NY, United States; ^2^ Shenzhen Hospital of Guangzhou University of Chinese Medicine, Shenzhen, China; ^3^ Carle Place Middle and High School, Carle Place, NY, United States; ^4^ Institute for Biotechnology, St. John’s University, Queens, NY, United States

**Keywords:** antimicrobial resistance, mechanisms of drug resistance, antibiotics, tolerance, multidrug-resistant

## Abstract

Once hailed as miraculous solutions, antibiotics no longer hold that status. The excessive use of antibiotics across human healthcare, agriculture, and animal husbandry has given rise to a broad array of multidrug-resistant (MDR) pathogens, posing formidable treatment challenges. Antimicrobial resistance (AMR) has evolved into a pressing global health crisis, linked to elevated mortality rates in the modern medical era. Additionally, the absence of effective antibiotics introduces substantial risks to medical and surgical procedures. The dwindling interest of pharmaceutical industries in developing new antibiotics against MDR pathogens has aggravated the scarcity issue, resulting in an exceedingly limited pipeline of new antibiotics. Given these circumstances, the imperative to devise novel strategies to combat perilous MDR pathogens has become paramount. Contemporary research has unveiled several promising avenues for addressing this challenge. The article provides a comprehensive overview of these innovative therapeutic approaches, highlighting their mechanisms of action, benefits, and drawbacks.

## 1 Introduction

Antibiotics are one of the most notable medical achievements of the 20th century. Their development in clinical area has revolutionized the approach to treating infectious diseases and has played a crucial role in saving countless lives suffering microbial infections (Ghosh et al., 2020; Chahar et al., 2023; Mehrotra et al., 2023; Upadhayay et al., 2023). The wide application of antimicrobial reagents was regarded as a true blessing for humanity, not only serving medicinal purposes but also finding application in various fields, including animal breeding, care, and production. In low-income areas, antibiotics have been utilized as preventive measures for decades, further showcasing their widespread impact on public health and agriculture. Despite the increased focus on antimicrobial resistance (AMR), the occurrence of multidrug-resistant (MDR) infections continues to escalate (Griffith et al., 2012; Durao et al., 2018; Xuan et al., 2023). Moreover, resistance to newly developed drugs is frequently observed shortly after their introduction. Unfortunately, the rate of discovering new antimicrobial drugs has significantly declined due to factors like low profitability and shifting priorities within the pharmaceutical industry (Aminov and Mackie, 2007). This situation further compounds the AMR crisis, presenting a significant challenge to public health and medicine.

Salvador Luria (1912–1991) made significant contributions to our understanding of bacterial resistance to viruses (phages) (NIH, 2023). His research demonstrated that bacterial resistance to phages is a heritable trait, passed down through genetic inheritance. The rise of antimicrobial resistance has significantly amplified the impact of infectious diseases, leading to increased studies of AMR.

The primary objective of this article is to emphasize the fundamental mechanisms underlying antimicrobial resistance. Additionally, it aims to outline the emergence and evolution of these mechanisms, and elucidate the factors that contribute to their enduring presence over time. By comprehending the evolutionary forces propelling antimicrobial resistance, there is potential to uncover fresh insights into strategies for addressing this substantial public health challenge.

## 2 An overview of antimicrobials and antimicrobial resistance

In 1928, Alexander Fleming’s discovery of penicillin marked the first successful use of an antibiotic to save soldiers’ lives for treating infectious diseases during World War II (Fleming, 1980). Since then, the discovery rate of new antibiotic classes has dramatically increased, such as vancomycin and methicillin (Sengupta et al., 2013). However, resistant strains to these antibiotics were reported a few years later since they were introduced, such as vancomycin and methicillin. In 1943, Luria and his colleagues demonstrated the genetic inheritance of bacterial resistance to viruses (phages), which was regarded as a milestone in understanding the replication mechanism of viruses and the resistance mechanism of bacteria. In 1960, the emergence of penicillin-resistant strains gradually became a pandemic concern. To address the problem, new β-lactam antibiotics were introduced in medical practices (Salam et al., 2023). However, during this time, bacterial strains began developing resistance to these antibiotics, leading to what is now known as the β-lactamase cycle. In 1961, the methicillin-resistant *Staphylococcus aureus* (MRSA) was observed in the United Kingdom (Ohlsen et al., 1998). Since then, MRSA has become widespread across the globe.

From 1960 to 1980, the pharmaceutical industry appeared to be generating a sufficient number of new antimicrobials. Nevertheless, fewer antibiotics were developed after this “golden period” as the industry changed their focuses in drug development. This decline, coupled with the rising antimicrobial resistance, has resulted in a limited pipeline of new antimicrobials (Parmar et al., 2018). Consequently, bacterial infections, due to growing prevalence and evolving multidrug resistance, have become global health challenges in clinical settings.

A recent database, the Comprehensive Antibiotic Resistance Database (CARD) (https://card.mcmaster.ca/), reveals the existence of 5,159 reference sequences. Although only 381 pathogens are relatively less, there is little relief from the situation. This is due to the slow pace at which the new generation of therapeutically useful antibiotics is reaching the market.

## 3 How antimicrobial resistance happens: intrinsic, acquired, and adaptive

Drug resistance can be classified into three categories: intrinsic resistance, acquired resistance, and adaptive resistance, which are determined by the way of resistance development.

### 3.1 Intrinsic resistance

Intrinsic resistance refers to resistance resulting from the inherent characteristics of microorganisms (Melander et al., 2023). Changes in glycopeptide in the bacterial cell envelope is one of the examples of intrinsic resistance in Gram-negative bacteria by altering the impermeability of the outer membrane of the bacteria (Christaki et al., 2020). The restriction entry of antibiotics, cause by the prorins proteins, contributing to its resistance (Bellido et al., 1992). Additionally, in *Proteus mirabilis*, *Serratia marcescens*, *Burkholderia* spp., *Yersinia* spp., the *pmrCAB* operons, encodes the PmrC, mediates polymyxin and modification the lipid increases bacterial resistance to the polymyxin B (Marceau et al., 2004; Poirel et al., 2017).

### 3.2 Acquired drug resistance

The acquired drug resistance is achieved through the transfer of genetic material by multiple mechanisms. It emerges *via* spontaneous mutations of microorganism or the obtain of new genetic material conferring drug resistance to the microorganism (Durao et al., 2018; Gonzalez-Villarreal et al., 2022). Three primary mechanisms for such gene transfer horizontally: transformation, transduction, and conjugation. Transformation is a process of DNA recombination where heterogeneous DNA fragments from a donor enter a microorganism and become and integrated part of its inheritance properties (Winter et al., 2021). Yet this natural transformability is limited to only a few bacterial species. Transduction also involves the transfer of genetic material. Unlike transformation, transduction occur between a bacteriophage and an infected bacterium through the action of a bacteriophage (a virus that infects bacteria) (Reygaert, 2018). Conjugation is one of the most significant mechanisms of gene transfer horizontally. This process requires direct physical contact between bacterial cells, during which genetic material is transferred. This transfer occurs through the formation of a sex pilus, a plasmid is transferred in the recipient bacterium. In a single conjugation event, multiple there can be a few drug resistance genes located at the plasmid passing through the recipient bacterium, as a result, the recipient bacterium obtains the multidrug resistance (Graf et al., 2019; Wang et al., 2019; Gonzalez-Villarreal et al., 2022).

### 3.3 Adaptive drug resistance

Adaptive drug resistance is characterized as the capacity of microorganisms to adapt reversibility and become resistant to one or more antibiotics in response to specific environmental signals. The drug resistance response to some environmental conditions, such as stress, growth state, pH, concentrations of ions, nutrient conditions, or exposure to sub-inhibitory levels of antibiotics. Adaptive drug resistance is temporary in nature, which is different from other types of drug resistance. (Sandoval-Motta and Aldana, 2016; D'Aquila et al., 2023). It enables bacteria to respond swiftly to antibiotic challenges, but once the inducing signal is no longer present, the bacteria typically revert to their original susceptibility to the antibiotics (Lazar et al., 2023).

## 4 Biological mechanism of drug resistance

**FIGURE 1:**
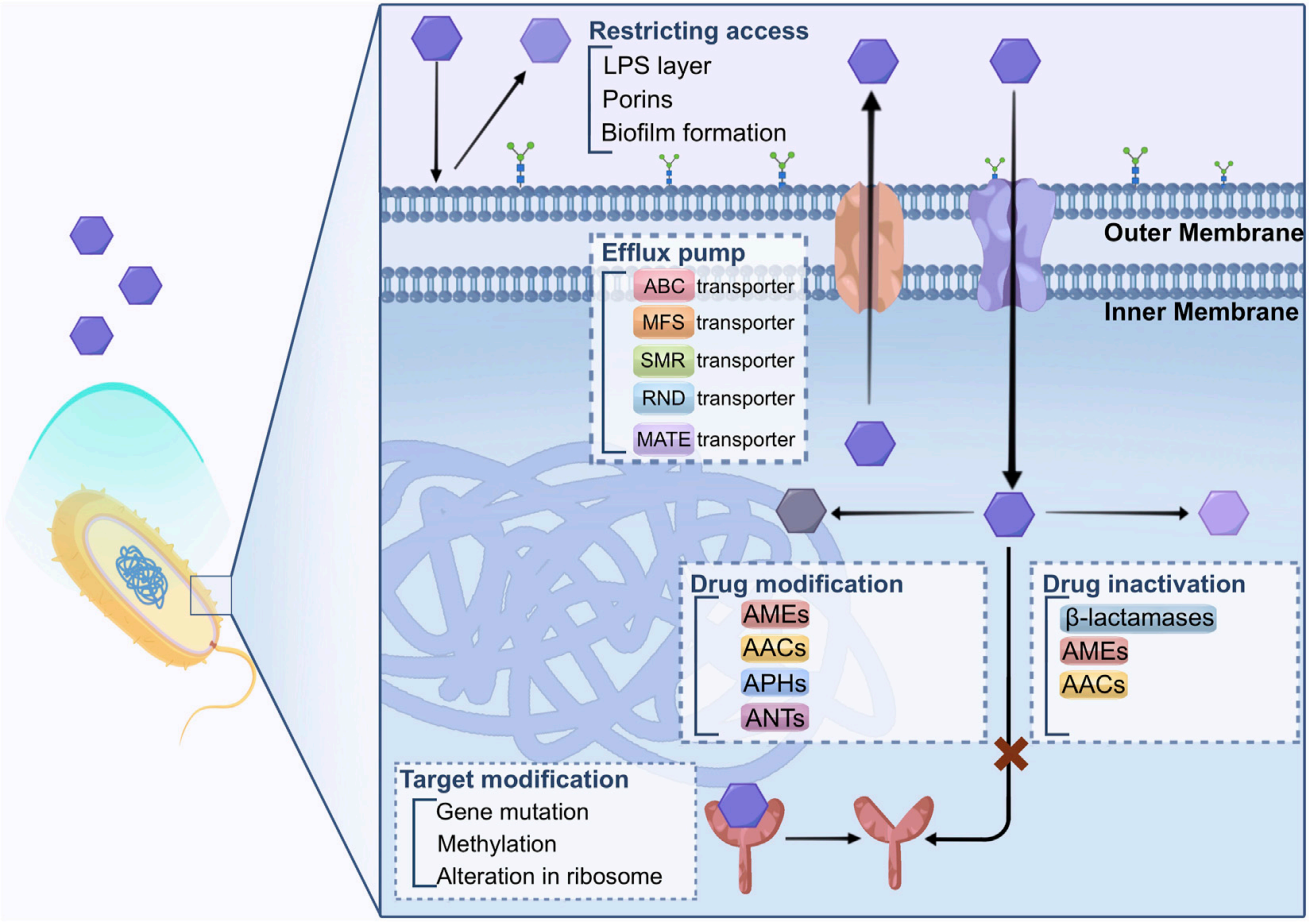
Biological mechanisms of drug resistance.

Bacteria have evolved several mechanisms to defend against the inhibition functions of antibiotics. The primary mechanisms that bacteria employ to develop drug resistance against antimicrobial agents involve limiting the restricting access of drugs, modifying the drug’s target, inactivating the drug, modifying the drug itself, targeting bypass, and enhancing active drug efflux from the cell. These strategies help microorganisms to withstand the effects of antibiotics, leading to the occurrence of AMR, as described in Figure 1.

### 4.1 Restricting access to drugs

There exists an inherent variation in the capacity of bacteria to restrict the entry of antimicrobial agents. The structures and functions of the lipopolysaccharide (LPS) layer in Gram-negative bacteria create a barrier against specific types of molecules. Consequently, these bacteria possess natural drug resistance to certain classes of prominent antimicrobial agents (Blair et al., 2014; Salam et al., 2023). Mycobacteria, on the other hand, have an outer membrane with a significant lipid content, making it easier for hydrophobic drugs like rifampicin and fluoroquinolones to penetrate the cell. However, the access to hydrophilic drugs is limited due to this hydrophobic barrier (Kumar and Schweizer, 2005; Baran et al., 2023).

The outer membrane of bacteria serves as a robust defense against undesirable substances. However, this protective barrier also hinders the entry of nutrient substances. To address this limitation, bacteria employ porins that enable the movement of hydrophilic molecules of certain sizes (Fernandez and Hancock, 2012).

Porins are beta barrel proteins that cross a cellular membrane and act as a pore, through which molecules can diffuse.

The porins are composed of a beta barrel structure that crosses cell membrane. The porins act as a pore with hydrophilic amino acids (AA) inside and hydrophobic AA locating at the position facing the outer membrane through which the molecule can pass and diffuse. This configuration creates a pore that facilitates the attraction and transportation of hydrophilic molecules entering the cell. While porins can exist as monomers, they are often found in stable trimers, and each unit is believed to function independently (Vergalli et al., 2020). Porins causing antibiotic resistance are associated with the decreased amount of porins or reduced porin protein expression.

In a clinical strain of *Klebsiella pneumoniae*, for example, a nonsense mutation in the OmpK36 porin cause the protein cannot translated correctly (Wozniak et al., 2012). However, the bacterium the development of compensated for this defect by up-regulating other selective porins, ensuring the maintenance of significance transportation of nutrient molecules. Such adaptation might lead to developing resistance to carbapenem antibiotics (Garcia-Fernandez et al., 2010). As a result, the penetration of antibiotics into the cell is limited, contributing to the bacterium’s innate resistance to antimicrobial agents.

The development of biofilms by certain microorganisms contribute to another mechanisms of resistance. Such biofilms are found in *Escherichia coli*, *Staphylococcus epidermidis,* and *Pseudomonas aeruginosa*. The feature of biofilm in organisms which are of great variety is the attachment to the surface *via* production of extracellular polymers. Biofilms can provide defense and attachment to bacterial cells with increased tolerance and resistance to antibiotics through various processes, including obstructing the penetration of antibiotics. The growth of biofilm-forming organisms is slower, this can be explained that limited by nutrient and oxygen can be utilized. Additionally, biofilms can reduce the of antibiotics across the biofilms, making it harder for antimicrobial agents to eliminate the bacteria within the biofilm effectively. As a result, bacteria in biofilms can evade the effects of antibiotics, leading to persistent infections that are challenging to the treatment (Van Acker et al., 2014; Hall and Mah, 2017; Dutt et al., 2022).

### 4.2 Target modification

Bacteria can alter in the target sites of antibiotics is a mechanism of resistance, making it difficult or even ineffective for drugs to bind to the altered target. This alteration is caused by gene mutations for the expression of protein that composes the drug target site.

For example, in DNA gyrase if there are mutation in quinolone-resistance-determining region (QRDR), it will contribute to resistant to fluoroquinolone in microorganism (Ashley et al., 2017). Methylation of genes can be another strategy for the development of AMR. By utilizing erm methylases against macrolides, lincosamides, and streptogramin B antibiotics in both Gram-positive and Gram-negative bacteria (Saha and Sarkar, 2021). Additionally, methylation of the *cfr* gene has been associated with drug resistance development in various bacteria. *Staphylococcus* spp. demonstrate a decreased affinity to β-lactam antibiotics because the change of in the penicillin-binding protein sites (Foster, 2017). These modifications in drug targets are crucial factors contributing to bacterial resistance against specific classes of antibiotics (Darby et al., 2023).

Alteration in the ribosome results in AMR that impacts protein expression, including macrolides, tetracycline, chloramphenicol, aminoglycosides (AGs), etc. Aminoglycosides are bound to the 30S subunit of ribosome, while chloramphenicol, macrolides, lincosamides, and streptogramin B antibiotics bind to the 50S subunit of ribosome, causing the inhibition of protein expression (Lambert, 2002; Kapoor et al., 2017; Tarin-Pello et al., 2022).

### 4.3 Drug inactivation

Three primary enzymes are responsible for inactivating antibiotics, namely, β-lactamases, aminoglycoside-modifying enzymes, and chloramphenicol acetyltransferases (AACs) (Kapoor et al., 2017; Reygaert, 2018).

Specific enzymes neutralize aminoglycoside modifying enzymes (AGEs) by carrying out phosphoryl-transferases, nucleotidyl transferases, adenylyl-transferases, and AACs reactions (Costa et al., 2020; Jannat et al., 2022). These aminoglycoside-modifying enzymes (AMEs) alter the structure of aminoglycoside molecules, reducing their affinity and hindering their binding to the 30S ribosomal subunit. Consequently, AMEs provide extended spectrum resistance to aminoglycosides (AGs) and fluoroquinolones (FQs) (Strateva and Yordanov, 2009). AMEs have been identified in strains *of S. aureus*, *Enterococcus faecalis*, and *Streptococcus pneumonia* (Kapoor et al., 2017; Ahmadian et al., 2021).

Resistance to chloramphenicol is found in certain Gram-positive and Gram-negative bacteria, as well as some *Haemophilus influenzae* strains. These resistant bacteria produce an enzyme called chloramphenicol acetyltransferase, which acetylates hydroxyl groups of chloramphenicol (Tolmasky, 2000). This modified form of chloramphenicol loses its ability to bind to the 50S ribosomal subunit properly (Schwarz et al., 2004).

β-lactamases, for instance, can hydrolyze a wide range of β-lactam antibiotics containing ester and amide bonds, such as penicillin, cephalosporins, monobactams, and carbapenems (Diene et al., 2023). To date, approximately 300 different β-lactamases have been identified. These enzymes are broadly distributed and can be classified using two central systems: Ambler (structural classification) and Bush-Jacoby-Medeiros (functional classification). Here, we discuss the widely used Ambler classification of β-lactamases.

In the beginning, Ambler categorized β-lactamases into two primary groups (Salahuddin et al., 2018; Tooke et al., 2019): class A, which encompasses serine β-lactamases with active sites, and class B, which consists of metallo-β-lactamases reliant on a divalent metal ion, usually Zn^2+^, for their function (Hall and Barlow, 2005). Subsequently, a novel form of serine β-lactamases was uncovered, displaying modest sequence similarity to the pre-existing class A enzymes. This newly identified group was labeled as class C or AmpC β-lactamases. Furthermore, another set of serine β-lactamases, known as OXA β-lactamases, emerged. These enzymes are unlike the other types β-lactamases, prompting their classification as class D.

### 4.4 Drug modification

Drug modification is a frequently utilized strategy to render antibiotics ineffective, particularly in the case of aminoglycosides (e.g., kanamycin, gentamicin, and streptomycin), chloramphenicol, and β-lactams. Several AMEs have been identified in producer bacteria, including N-acetyl transferases (AAC), O-phosphotransferases (APH), and O-adenyltransferases (ANT) (Peterson and Kaur, 2018). These enzymes modify aminoglycoside antibiotics by acetylating, phosphorylating, or adenylylating them. The *Streptomyces* species produce these enzymes that show similar biological activities to the antibiotic-resistant clinical strains. But the connection between modification enzymes and the biosynthesis of aminoglycoside in the host is not clear (Benveniste and Davies, 1973). Certain species may harbor enzymes responsible for modification despite not producing antibiotics, and *vice versa*.

Streptomycin resistance is an exception as both antibiotic biosynthesis pathway and related enzyme responsible for modification in self-resistance are understood. For example, self-resistance of streptomycin in *Streptomyces griseus* is achieved by the 6-phosphotransferase that can transfer the active product to an inactive form streptomycin-6-phosphate (Christaki et al., 2020). The 6-phosphotransferase is involved in the last step in the streptomycin synthesis, and its production is affected by genes involved in synthesis pathway.

However, biological activities of the AMEs in the host bacteria are controversial (Munir et al., 2023). Some believe that AMEs are not involve contribute to resistance in host bacteria while might serve other metabolic functions (Martinez, 2018; Lu et al., 2023). The sequence analysis of AMEs demonstrated the diversity of these enzymes and are encoded by different genes. Since they have similar biological functions, they may experience different convergent paths for similarity. Indeed, these enzymes have certain sequence and structural similarity between AMEs and other cell metabolic proteins (Saleh et al., 2023).

### 4.5 Drug efflux

Efflux pumps, responsible for extrusion of drugs and toxins from bacterial cells, are bacterial transport proteins that care classified into two types, primary and secondary transporters (Blair et al., 2014; Alenazy, 2022; Salam et al., 2023). The primary transporters belong to the ATP-binding cassette (ABC) family and activated via ATP binding and hydrolysis to facilitate efflux. On the other hand, the secondary transporters include various families, including the major facilitator superfamily (MFS), resistance nodulation division (RND) family, small multidrug resistance (SMR) family, and multidrug and toxic compound extrusion (MATE) family (Reygaert, 2018; Hajiagha and Kafil, 2023; Palazzotti et al., 2023).

These secondary transporters rely on the energy generated by the electrochemical potential of the membrane to drive the efflux process. For example, a recent study indicated that GI-M202a, in conjunction with the MFS transporter in *Pseudomonas pnomenusa*, played a crucial role in facilitating the transmission of polymyxin B resistance (Gao et al., 2023). The regulatory mutations result in the increased expression of efflux pumps and can cause MDR in the bacteria (Huang et al., 2022; Lorusso et al., 2022). This phenomenon has been predominantly observed in efflux systems belonging to the RND efflux family (Colclough et al., 2020). These regulatory mutations can enhance the efflux activity of these pumps, allowing the bacteria to expel multiple types of drugs and thereby develop resistance to various antibiotics. In cancer cells, MDR-related drug transporters, such as ABC transporters, are active in the efflux of drugs, conferring MDR to cancer cells (Banerjee et al., 2023; Bo et al., 2023; Fan et al., 2023).

*Acinetobacter baumannii* is an MDR pathogen commonly found in healthcare settings, where choices for treatment are frequently restricted. The overexpression of AdeABC, one of the multidrug RND efflux pump is responsible for the drug resistance of *A. baumannii* (Colclough et al., 2020). The increased number of RND efflux pumps causes the *A. baumannii* resistant to an extensive variety of antibiotics, such as aminoglycosides. It reduces susceptibility to fluoroquinolones, tetracycline, tigecycline, chloramphenicol, erythromycin, trimethoprim, netilmicin, meropenem, and even the dye ethidium bromide. Studies of clinical MDR strains of *A. baumannii* have demonstrated that the primary contributor to drug resistance is often the AdeABC efflux pump. This significantly constrains the potential treatment choices against this microorganism (Ragueh et al., 2023). Consequently, tigecycline is regarded as the last-resort antibiotics for addressing infections brought about by MDR Gram-negative bacteria (Hornsey et al., 2011).

## 5 Fight back against drug resistance

### 5.1 Detection of drug resistance/tolerance

Drug resistance/tolerance can be assessed using killing assays like time-kill curves, which display a bimodal killing pattern in cases of drug persistence (Foerster et al., 2016). However, these assays are labor-intensive and show significant variability, necessitating numerous repetitions and making them less feasible for routine clinical microbiology. Moreover, the levels of antibiotic persistence observed *in vitro* in laboratories might not accurately reflect the persistence levels in patients from whom the bacterial strains were isolated, considering the complex factors contributing to this unstable phenomenon. Consequently, measurements of antibiotic persistence in laboratory conditions only offer approximations of bacterial behaviors during *in vivo* infections. Nonetheless, they still provide essential supplementary information to aid physicians in making therapeutic decisions (Huemer et al., 2020).

An alternative method for testing drug tolerance is the “Replica Plating Tolerance Isolation System” (REPTIS), developed by Hiramatsu and colleagues, which has proven successful in identifying and selecting ciprofloxacin (CIP)-tolerant mutants in *S. aureus* (Matsuo et al., 2019). REPTIS does not require adjusting antibiotic concentrations, addressing this limitation. In this method, a sterile silk cloth transfers colony-forming units (CFUs) onto a fresh plate (replica plate) where surviving bacteria can grow. The level of drug tolerance is determined by counting the number of growing bacteria within the former inhibition zone. While REPTIS cannot detect induced antibiotic persistence, it holds the potential for adaptation in automated use within diagnostic microbiology laboratories.

The MALDI-TOF MS-based approaches are utilized for the efficient antimicrobial resistance identification (Florio et al., 2020). This technology has been investigated for detecting antimicrobial resistance in pathogenic fungi (Florio et al., 2018). The aim is to address the urgent need for identifying drug resistance patterns quickly and accurately, allowing clinicians to make informed treatment decisions for better patient outcomes. A detection protocol using MALDI-TOF MS was explicitly studied for Carbapenemase-Producing Organism (CPO), *Bacteroides fragilis*, a Gram-negative strain carrying the drug resistant gene *cfiA* that can encode the carbapenemase enzyme (Johansson et al., 2014).

### 5.2 Strategies against drug resistance/tolerance

**FIGURE 2:**
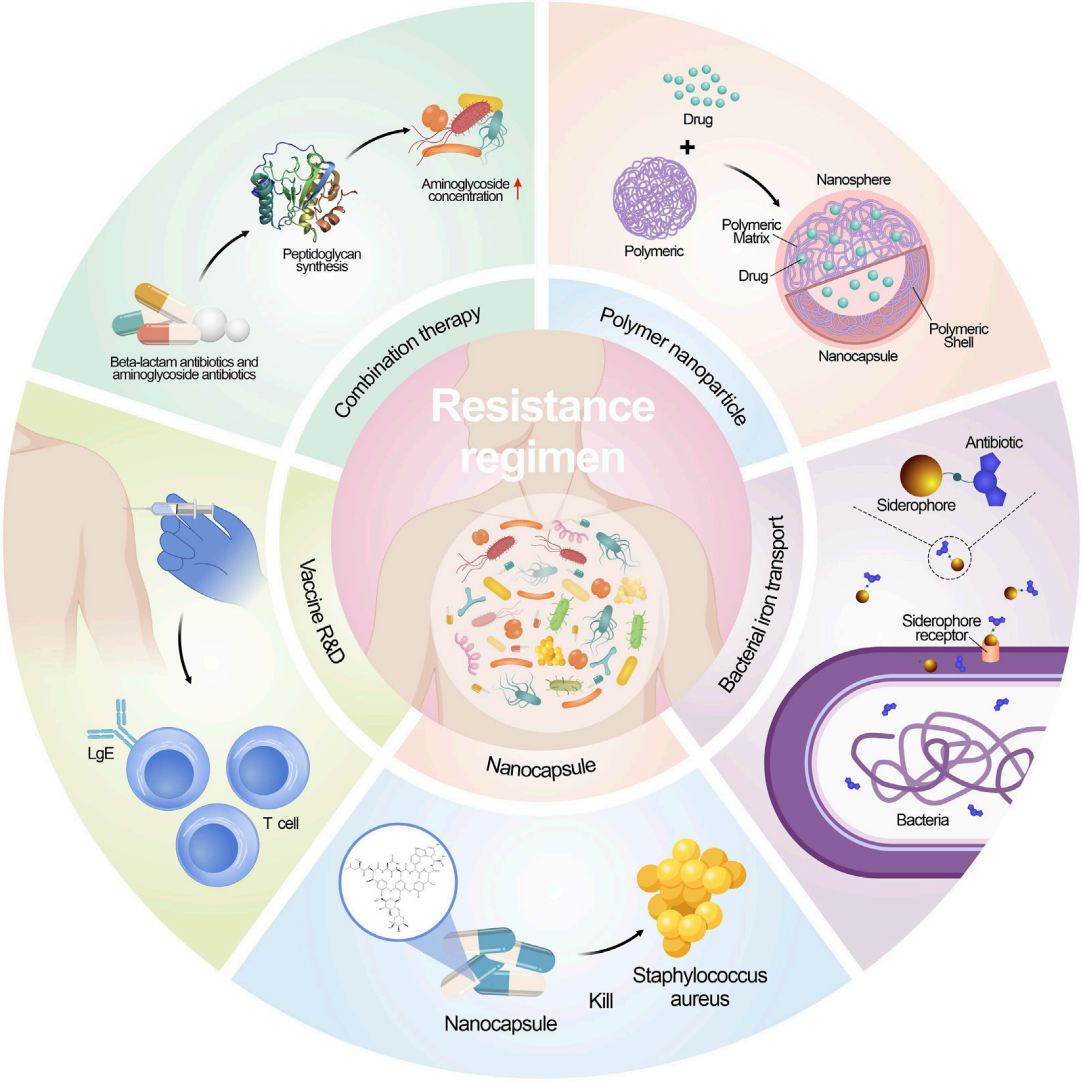
Strategies implemented to address microbial drug resistance.

Antimicrobial resistance is a crucial global health challenge, and unfortunately, there is no straightforward remedy (Morrison and Zembower, 2020; Kaur et al., 2022; Talaat et al., 2022). Current endeavors revolve around enhancing diagnosis, antibiotic-prescribing methods, and infection prevention strategies to combat this issue. Nevertheless, the development of new antimicrobial compounds has been limited, and those under consideration often do not belong to new antibiotic classes (Spizek, 2018). Additionally, the effectiveness of new antimicrobials is compromised by the rapid adaptability of microorganisms, leading to their potential short lifespan. Consequently, innovative treatment approaches are indispensable in the battle against both existing and evolving antimicrobial resistance (Blair et al., 2014; McEwen and Collignon, 2018). Figure 2 shows the contemporary strategies employed to combat microbial resistance.

#### 5.2.1 Combination therapy

Traditionally, the combination of antibiotics has been utilized in the treatment of MDR microbial. Among these combinations, β-lactam antibiotics are particularly notable due to their broad-spectrum activity and ability to synergize with other kinds of antibiotics. The combination of a β-lactam antibiotic along with an aminoglycoside antibiotic has been extensively applied to combat infections caused by the Gram-negative bacteria (Murugaiyan et al., 2022). The mechanism behind this synergy lies in the β-lactam antibiotics’ ability to impair peptidoglycan synthesis, which results in an increased intracellular concentration of aminoglycosides within the bacterial cell. It has been long believed that the combination treatment of antibiotics can be more effective than using only one antibiotics, yet the outcomes are not always ideal due to the continuous evolve of resistance mechanisms (Tamma et al., 2012). As a result, it is crucial to continuously assess the efficacy of these antibiotic pairs and any emerging alternative strategies in the context of multidrug environments to combat AMR effectively (Hegreness et al., 2008; Yu et al., 2019). Regular evaluation and adaptation of treatment approaches are essential in the ongoing fight against AMR.

The clinical success in reducing the emergence of resistance through combination therapy is well illustrated in the case of tuberculosis (TB) treatment. The first documented use of antibiotic combination therapy for TB involved streptomycin and para-amino salicylic acid with the therapeutic effects and reduction of resistance (Kerantzas and Jacobs, 2017). Currently, the World Health Organization (WHO) recommends a 6–9 months’ combination therapy for TB, involving drugs that target different metabolic pathways. Drugs include isoniazid, rifampicin, ethambutol, pyrazinamide, etc. (Kuck et al., 1963; Wehrli, 1983; Unissa et al., 2016; Nusrath Unissa and Hanna, 2017). Despite the extensive use of combination therapy, TB resistance has still emerged. This is attributed to poor adherence to the lengthy and challenging treatment regimens, which are both costly and difficult to implement. To address this issue, there is a need for new drugs and drug combinations that can effectively control the disease while reducing the duration of the 6–9 months’ chemotherapy.

Adverse effects in drug combinations can arise from both pharmacokinetic and pharmacodynamic interactions. Pharmacodynamic interactions occur when drugs directly influence each other’s effects, and these interactions can be either synergistic or antagonistic (Coates et al., 2020). Additionally, pharmacodynamic effects may extend beyond the target bacteria, leading to unintended effects elsewhere in the body. On the other hand, pharmacokinetic interactions impact the absorption, distribution, metabolism, and elimination of drugs, resulting in alterations in effective concentrations in blood and tissues (Sinel et al., 2017). This is significant because exposure to sub-inhibitory concentrations of antibiotics can hasten the development of resistance. Recent data have linked the combination of vancomycin and piperacillin–tazobactam (common empirical hospital treatments) to acute kidney injury (AKI) (Scully and Hassoun, 2018). The probability of developing AKI with this combination was higher compared to either vancomycin or piperacillin–tazobactam used as monotherapy (27.66% vs. 6.98% or 7.92%, respectively). This emphasizes the importance of detecting and assessing toxicity in combination therapy.

#### 5.2.2 Development of vaccines

New strategies to inhibit the growth of resistant bacteria, including developing novel antibiotics. Yet discovering new chemical compounds with optimal biological activity, pharmacokinetics, pharmacodynamics, metabolism, and biosafety is a formidable mission (Rosini et al., 2020). Consequently, vaccines are emerging as valuable and effective weapons in the fight against AMR. A significant advantage of vaccination is that the drug resistant mechanisms are less problematic than antibiotics. As mentioned earlier, antibiotic resistance arises through intrinsic mutations or obtain mobile genetic elements through horizontal gene transfer, enabling bacteria to survive the killing effects of drugs. Furthermore, vaccines offer multiple targets while most antibiotics offer a single target. Consequently, the commence of microbial resistance to vaccine can be challenging as more mutations are needed to occur (Tagliabue and Rappuoli, 2018). Gene engineering has been utilized to create specific vaccines, which involve administering specific antigenic determinants to confer protection without cause significant health effects to the vaccinated patients. Such approach has been successfully utilized to develop the human rotavirus vaccine and vaccine that is live influenza attenuated. Focusing on vital antigenic components, these vaccines effectively stimulate the immune system to generate protective responses against the targeted pathogens while minimizing potential adverse effects on the vaccinated subjects (Finco and Rappuoli, 2014; Nogales and Martinez-Sobrido, 2016; Rosini et al., 2020; Jingshu Yang and Yang, 2022).

Vaccines can effectively combat AMR through decreasing the inappropriate use of antimicrobial compounds. For instance, viral vaccines targeting the influenza virus can reduce the incidence of fever and illness among a considerable portion of the elderly population residing in communities in the US (Nichol et al., 2007). Interestingly, vaccines reduce the inappropriate use of antibiotics caused by viral infections.

Vaccines contribute to the reduction of resistant serotypes. Pneumococcal polysaccharide conjugate vaccines, for example, led to a decrease in antibiotic prescriptions and therefore reduced the prevalence of antibiotic-resistant strains (Moore et al., 2015). However, there are limitation in application of vaccines. Nevertheless, there remains a high risk of the evolution of antimicrobial resistance in pneumococcal serotypes not covered by the vaccine (Rosini et al., 2020). In the 1990s, the 7-valent pneumococcal conjugate vaccine (PCV7) led to an increased prevalence of serotype 19A, a non-vaccine serotype with a high rate of penicillin resistance. The introduction of the 13-valent PCV in 2010, containing six additional serotypes, including 19A, further decreased the incidence of IPD and antibiotic-resistant *pneumococci* (Gaviria-Agudelo et al., 2017).

#### 5.2.3 Optimizing drug delivery systems

Antibiotic research is confronted with a significant hurdle - the limited cell permeability of antibiotics. To address this issue, the promising delivery systems have been developed to facilitate the drug to enter the cell (Li et al., 2023). A critical strategy in overcoming antibiotic resistance is to exploit better the transport systems, such as the synthetic siderophore derivatives improve the entry of antibiotics. A notable study showed a conjugate containing ampicillin demonstrated remarkable results. The conjugate exhibited a 100-fold increase against Gram-negative enterobacteria compared to using ampicillin alone and showed 1000-fold increase in inhibiting the growth of *P. aeruginosa* (Mollmann et al., 2009). Polymeric nanoparticles have emerged as a promising strategy to address several challenges in antimicrobial therapy. A different type of nanocapsule was shown to contain hydrophilic polymersomes with encapsulated vancomycin, which enhanced the treatment efficacy, particularly against infections caused by methicillin-resistant *S. aureus*. The nano-emulsification system enhances drug solubilization in water and improves bioavailability by creating smaller particles that increase the surface area for absorption. This system allows for better drug dispersion, leading to increased solubility and more efficient absorption in the body. A self-nanoemulsifying preconcentrate (EB-P) of ebselen was prepared, exhibiting more potent anti-fungal activity against azole resistance strain *Candida albicans* (Vartak et al., 2020; Menon et al., 2021).

#### 5.2.4 Utility of artificial intelligence (AI)

As mentioned before, numerous pathogenic bacteria have exhibited a rising trend of resistance to existing antibiotics, while developing new antibiotics has been significantly limited. Using an AI algorithm can be a novel way to accelerate the drug discovery process. The researchers trained a neural network and screened approximately 7,500 molecules. A compound named abaucin was discovered effectiveness in controlling an *A. baumannii* infection (Liu et al., 2023).

AI, especially machine-learning (ML) and deep-learning (DL) techniques, is not only applied in the design of new antibiotics, but also utilized in creating synergies through combinations of drugs (Hu et al., 2019). The machine learning algorithms analyze patterns to analyze AMR assist healthcare providers and policymakers in making decisions as it can predict the resistant bacteria/fungal via development of resistance to certain drugs or compounds (Rabaan et al., 2022; Amsterdam, 2023). In addition, machine-learning models can be utilized as the surveillance of AMR by analyzing data on antimicrobial use and resistant micro-organism, they can aid public health authorities to make informed decisions, prepare and respond immediately in the outbreak of resistance health issue when these models identify and predict the identify emerging resistance patterns and potential population and areas (Rabaan et al., 2022). These applications contribute to reducing the overall burden of AMR (Ali et al., 2023).

#### 5.2.5 Other strategies

Scientists are diligently exploring natural resources in search of potential alternatives to antibiotics. Plants are recognized as a great resource of antimicrobial agents (Abd El-Hack et al., 2022; Al-Amin et al., 2022). A variety of compounds originated from plants with potent antimicrobial activities, such as alkaloids, polyphenolics, flavonoids, and certain plant extracts (Islam et al., 2021; Kundo et al., 2021; Foyzun et al., 2022; Pawar et al., 2022). Although several phytochemical compounds have been identified in the research, there remain numerous compounds that require further investigation. The challenge lies not only in identifying these valuable discoveries but also in effectively translating them from laboratory research into practical applications within hospitals and clinical practices. Transferring these natural antimicrobial agents into real-world healthcare settings presents a significant hurdle that researchers are working to overcome (Gupta and Sharma, 2022; Salam et al., 2023).

Another strategy is to increase the effective concentration of antimicrobials within bacterial cells. This can be achieved through potentiation, wherein non-essential bacterial components, like efflux pump inhibitors (Lamut et al., 2019), are manipulated, or by using membrane transporters, e.g., iron transporters, *via* bound to an iron-binding siderophore mimetic group to facilitate antibiotics to enter and exert biological functions. A recent study identified di-berberine conjugates that exhibit enhanced synergistic effects with aminoglycosides. This highlights a valuable probe and its potential as lead compounds in developing efflux pump inhibitors (EPIs).

## 6 Conclusion

The development of chemical compounds with antimicrobial activities since the 19th century facilitated the commencement of antimicrobial therapy era. The escalation of antibiotic resistance and the upsurge in challenging-to-treat infections have driven extensive investigations since the early 20th century (Chen et al., 2022). Prof. Luria and his colleagues showed the mechanism of bacterial resistance to viruses. Later, more innovative mechanisms and related antimicrobial strategies were introduced. Recent advancements in antibiotic development offer optimism for fresh avenues in treating infections triggered by extensively resistant bacteria. Nonetheless, the pressing demand for ongoing research and discovery in antibiotics remains urgent, particularly to counter the impending post-antibiotic era (Sannathimmappa et al., 2021; Shariati et al., 2022).

The mechanisms outlined in this discussion exhibit a diversity that parallels the range of bacteria themselves. These bacterial defense mechanisms encompass a broad spectrum of antimicrobial agents at our disposal, and additional resistance mechanisms exist which have yet to be identified. Given this complexity, the prospects for combatting microorganisms might appear somewhat challenging.

Combating AMR requires a globally unified approach, involving close coordination between international governmental and nongovernmental agencies, underpinned by robust political support. Success hinges on integrating and collaborating across diverse research fields such as innovative resistance detection methods, combination therapy, vaccine development, efficient drug delivery systems, and artificial intelligence. This joint effort aims to effectively counteract the ongoing trends of AMR, with the goal of reducing its impact on both health and economies.
